# Quantifying ecosystem service trade‐offs for plantation forest management to benefit provisioning and regulating services

**DOI:** 10.1002/ece3.3286

**Published:** 2017-08-28

**Authors:** Er‐fu Dai, Xiao‐li Wang, Jian‐jia Zhu, Wei‐min Xi

**Affiliations:** ^1^ Key Laboratory of Land Surface Pattern and Simulation Institute of Geographic Sciences and Natural Resources Research Chinese Academy of Sciences Beijing China; ^2^ University of Chinese Academy of Sciences Beijing China; ^3^ National Marine Data and Information Service Center Tianjin China; ^4^ Department of Biological and Health Sciences Texas A&M University Kingsville TX USA

**Keywords:** ecosystem service, forest management strategies, Gan River Basin of South China, trade‐off/synergy

## Abstract

There is increasing interest worldwide regarding managing plantation forests in a manner that maintains or improves timber production, enhances ecosystem services, and promotes long‐term sustainability of forest resources. We selected the Gan River Basin, the largest catchment of Poyang Lake and a region with a typical plantation distribution in South China, as the study region. We evaluated and mapped four important forest ecosystem services, including wood volume, carbon storage, water yield, and soil retention at a 30 × 30 m resolution, then quantified their trade‐offs and synergies at the county and subwatershed scales. We found that the wood volume and carbon storage services, as well as the soil retention and water yield, exhibited synergistic relationships. However, the carbon storage displayed a trade‐off relationship with the water yield. Additionally, we compared the beneficial spatial characteristics among dominant species in the study region. The results showed that the Chinese fir forest and the pine forest exhibited lower overall benefits than natural forests including the broad‐leaved forest and the bamboo forest. To propose a suitable management strategy for the study region, method of spatial cluster analysis was used based on the four eco‐services at the subwatershed scale. The basin was divided into four management groups instead of treating the region as a homogenous management region. Finally, we proposed more specific and diverse management strategies to optimize forest benefits throughout the entire region.

## 1 INTRODUCTION

Ecosystem services are the benefits humans receive from the natural processes and structures of ecosystems. Ecosystem services are closely related to human well‐being (Costanza et al., 1997; Daily, 1997; Tilman, Cassman, Matson, Naylor, & Polasky, 2002). In recent years, with more thorough understanding of ecosystem service interactions, there has been growing evidence that management options may lead to beneficial trade‐offs or synergies between different ecosystem services, and these relationships are often highly nonlinear (Barbier et al., 2008; Carpenter et al., 2006; Chan, Shaw, Cameron, Underwood, & Daily, 2006; Lester et al., 2013). In many situations, attempts to optimize a single service often lead to reductions or losses of other services, as results of trade‐off among these services. Therefore, knowledge and awareness of interactions between ecosystem services are necessary for making sound decisions regarding appropriate management of natural systems and achieving maximum profits (Faith et al., 2010; Prato, 2012; Smith et al., 2013; Vidal‐Legaz, Martínez‐Fernández, Picón, & Pugnaire, 2013).

As a key component of terrestrial ecosystem, forest ecosystem plays an irreplaceable role in providing multiple services and products (e.g., biodiversity, carbon sequestration, water yield, and timber) for human society (Cademus, Escobedo, McLaughlin, & Abd‐Elrahman, 2014; Chisholm, 2010; Grasso, 1998; Onaindia, Fernández de Manuel, Madariaga, & Rodríguez‐Loinaz, 2013). According to the global forest resources assessment by the Food and Agriculture Organization of the United Nations (FAO) in 2010, the world's total forest area is just over 4 billion hectares. Among the global forest resources, close to 1.2 billion hectares (30%) are managed primarily for the production of wood and nonwood forest products, and an additional 949 million hectares (24%) are designated for multiple services, generally including the production of wood and nonwood forest products. In summary, the demands for protective or socioeconomic functions provided by forests are increasing, resulting in intensive management and a partial shift in designation from production to multiple services (Food and Agriculture Organization of the United Nations, 2010). Natural forests are affected by human activities throughout the history, and planted forest extensions have dramatically increased throughout the world (Hanowski, Niemi, & Christian, 1997; Lamb, 1998). The total area of global planted forest is estimated to be 264 million hectares (7%), increasing by approximately 5 million hectares per year (Food and Agriculture Organization of the United Nations, 2010). Furthermore, planted forests contributed to approximately 2/3 of global round‐wood production, reflecting an increased reliance on planted forests for wood production (Farley, 2007; Food and Agriculture Organization of the United Nations, 2010; Kanowski, Catterall, & Wardell‐Johnson, 2005). Thus, planted forests will increasingly contribute to supplying the world's wood and fuel, as well as to protecting soil and water resources, and this shift will help reduce the pressures on natural forests (Food and Agriculture Organization of the United Nations, 2010). However, land‐use change and plantation expansion have created unprecedented spatial patterns for natural resources at the global and regional scales, and the imbalance between different types of ecosystem services has led to urgent and widespread social demands for scientific forest management. In particular, as regional environmental problems such as global climate change, biodiversity loss, and water pollution become increasingly apparent, forest managers are seeking regional‐level management options that can be applied over space and time to mitigate environmental pressures and improve long‐term human well‐being. Thus, forest management options that can continue to provide economically valuable goods or services and promote a good living environment will be strongly attractive. Due to differing spatial scales, trade‐offs or synergistic relationships between the same forest ecosystem services will differ (Bennett, Peterson, & Gordon, 2009; McNally, Uchida, & Gold, 2011; Meehan et al., 2013; Turner, Odgaard, Bocher, Dalgaard, & Svenning, 2014). Quantifying and comparing these relationships between forest ecosystem services at different spatial scales will help to determine the most effective and reasonable management units and adjust the spatial distributions of forests to minimize costs and undesirable results, as well as acquire the largest benefits and the best balance of forest ecosystem services (Bai, Zheng, Ouyang, Zhuang, & Jiang, 2013; Egoh, Reyers, Rouget, Bode, & Richardson, 2009; Egoh, Reyers, Rouget, & Richardson, 2011).

Currently, China has the largest area of and the fastest growing planted forests in the world. The total area of planted forests in China is 77 million hectares, accounting for 30% of the world's planted forests and increasing by approximately 1.4 million hectares per year through large‐scale afforestation (Almanac of China paper industry, 2010; Food and Agriculture Organization of the United Nations, 2010). Therefore, planted forests in China have played an increasingly important role in regional wood production, natural resources and biodiversity protection, socioeconomic development, and even improvement of forestry development in the Asian and Pacific regions. However, characteristics like weak ecological protection functions or lower internal ecological safety than that of natural forests are found in some planted forests, that is, there are often obvious beneficial trade‐offs between provisioning services and regulating services in planted forests (Calvino‐Cancela, Eugenia Lopez de Silanes, Rubido‐Bara, & Uribarri, 2013; Deal, Hennon, O'Hanlon, & D'Amore, 2014; Lu, Fu, Jin, & Chang, 2014). According to the Chinese forest resources assessment in 2010, the average wood volume of planted forests in China was 49.01 m^3^ per hectare, which is only 57% of the average wood volume of natural forests and 45% of the average wood volume of the world's forest. Therefore, low productivity of planted forest in China is still prominent, and a large gap remains compared to the productivity of developed countries (Zhang, Guan, & Song, 2012; Zhao et al., 2012). Analyze the spatial characteristics and interactions between the provisioning services and regulating services of the planted forest can provide information on their contributions to native economic benefits, increasing landscape connectivity and protecting populations at the regional scale (Carpenter et al., 2006; Chan et al., 2006; Egoh et al., 2009; Egoh et al., 2011). Moreover, it will also shed a scientific light on the cultivation and management intensification of artificial forests, guiding new forest management strategies to encourage the planted forest development with better synergies between wood production and ecological benefits (Bai et al., 2013; Egoh et al., 2011; McNally et al., 2011; Meehan et al., 2013).

The red soil hilly region in South China is an important wood production region, encompassing a total area of 1,180,000 km^2^, of which approximately 308,000 km^2^ is the planted forest, with the Chinese fir (*Cunninghamia Lanceolata*) and the Masson Pine (*Pinus Massoniana*) are the primary fast‐growing tree species for wood production. There has been a long history of the Chinese fir plantation cultivation in this region since the Tang and Song Dynasties (about 1,000 years ago), and forming a “production‐transport‐sale” model that woods carried along the river to the foothills based on the regional hilly conditions to support social and economic development (Sheng, 2014). However, plantation area of the Chinese fir and the Masson pine has rapidly increased since the foundation of new China (in 1949) in response to a call for “fast‐growing and high‐yield plantations” to meet the timber demand (Leng, Du, & Wang, 2007; Xie, 1992). A sharp increase of plantation areas led to a decrease in native forests and regional biodiversity, and furthermore fragmented background landscapes and environmental factors, especially those related to water and soil cycling. And an unreasonable “high‐intensity harvest” management strategy over multiple decades finally resulted in serious soil erosion problems and regional ecological imbalance (Ding & Qiu, 2001; Zhao et al., 2011; Zhu, Zhan, Yang, Hu, & Gu, 2009). In return, due to nutrients loss and management ignorance, large areas of remnant or low‐productivity plantations remain after harvesting. Thus, it is important and urgent to change the patterns and management strategies of plantation forests in this region to benefit provisioning and regulating services.

The aim of the study was to present a scientific and reasonable management strategy for the planted forests in the red soil hilly region in South China based on the spatial characteristics of ecosystem services and trade‐offs at the regional scale. Therefore, four main forest ecosystem services, including carbon storage, wood volume, water yield, and soil retention, were quantified and mapped using the InVEST model and CASA model combined with GIS software. Further trade‐offs or synergies between the four ecosystem services were analyzed and calculated using different spatial units to illustrate the scale effect of ecosystem services. Finally, we classified the typical subregions for forest management according to similar patterns in the spatial characteristics of ecosystem service productivity in order to benefit the provisioning and regulating services.

## 2 MATERIAL AND METHOD

### 2.1 Study area

**Figure 1:**
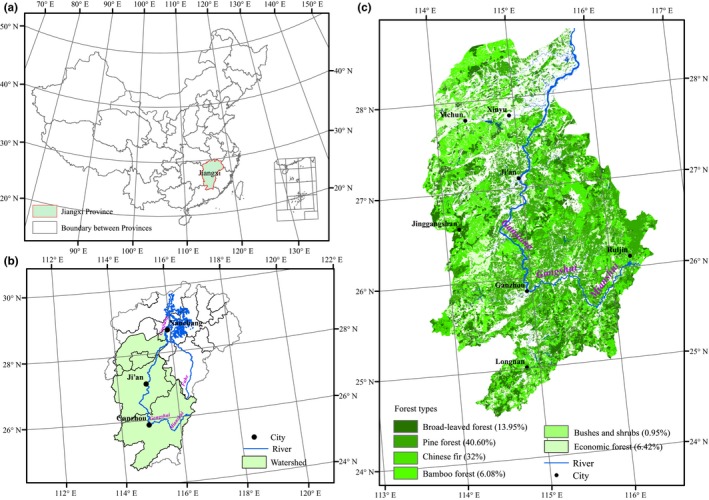
Location and different forest types in the Gan River Basin. (a) location of the Jiangxi Province in China; (b) location of the Gan River Basin in the Jiangxi Province; c, distribution of different forest types in the Gan River Basin

The study was conducted in the Gan River Basin (24°29′‐28°42′N, 113°42′‐116°38′E) (GRB, Figure 1), which is a typical plantation region in the red soil hilly region in South China. The Gan River, which is 823 km long and one of the eight major tributaries of the Yangtze River, flows northward into Poyang Lake near the city of Nanchang. The GRB is located in southwestern Jiangxi Province, China, occupying an area of 83,500 km^2^, in which the forested area is 6.11 × 10^4^ km^2^, and the population is approximately 20,000,000 (China statistical yearbook, 2010). The GRB has complex geomorphology, ranging from high to low then to high from east to middle, then to west and gradually tilting from south to north. The basin mainly comprises hilly areas, occupying 64.7% of the total area. The economic activity is essentially based on metallurgy, hydropower, and the development of local natural resources, particularly timber and forestry by‐products. The GRB is characterized by a subtropical monsoon climate. The average temperature is 17.8–19.7°C, and the seasonal and annual rainfall distributions are uneven. Less rainfall occurs in autumn and winter, while more occurs in spring and summer, averaging 1,341–2,207 mm annually. The relative humidity is 75%–83%. The zonal soil type in the GRB is mountain red soil, which is mainly distributed in regions below 600 m and is vulnerable to wind erosion. In addition to red soil, yellow, yellow–brown and mountain meadow soils can be found at higher elevations (Lin, 1986; Zhou, Wan, & Zheng, 2012).

The zonal vegetation in the GRB is characterized as subtropical evergreen broad‐leaved forest, and some of dominate dominants are *Schima superba, camphor, Cyclobalanopsis glauca, Castanopsis fargesii*. Due to the overuse of forest resources and the “high‐intensity harvesting” management strategy during the early twentieth century, natural broad‐leaved forests have rapidly disappeared, and planted forests dominated by the Chinese fir or the Masson pine have largely increased. Other typical and important forests are dominated by *dominants like Phyiiostachys pubescens, Juniperus, Liquidambar, Camellia*.

### 2.2 Quantification of ecosystem services

**Figure 2:**
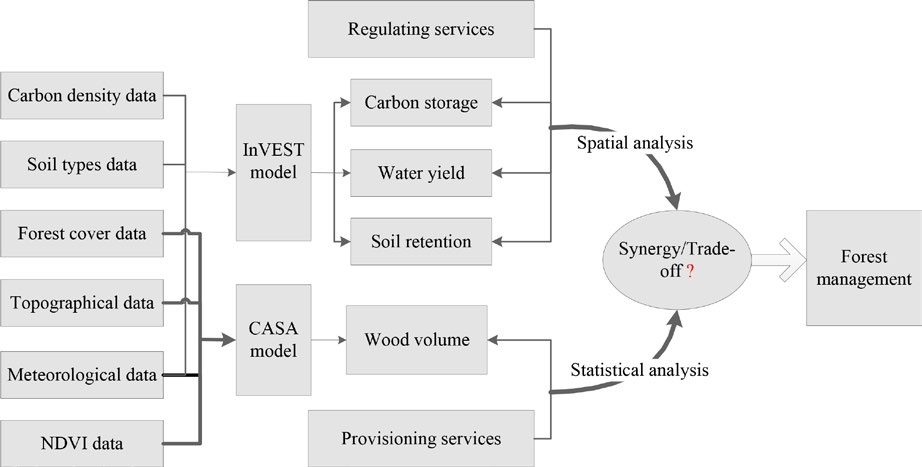
Flowchart of the forest ecosystem service analysis in the Gan River Basin

An integrated approach (Figure 2) utilizing the InVEST model, CASA model, and Geographic Information Systems (ArcGIS) software was used to quantify and map the main forest ecosystem services. The InVEST model is widely used for ecosystem service evaluation. It quantifies ecosystem services by employing a production function approach and specifies ecosystem service outputs based on the environmental conditions and processes (NCP, 2014).

**Figure 3:**
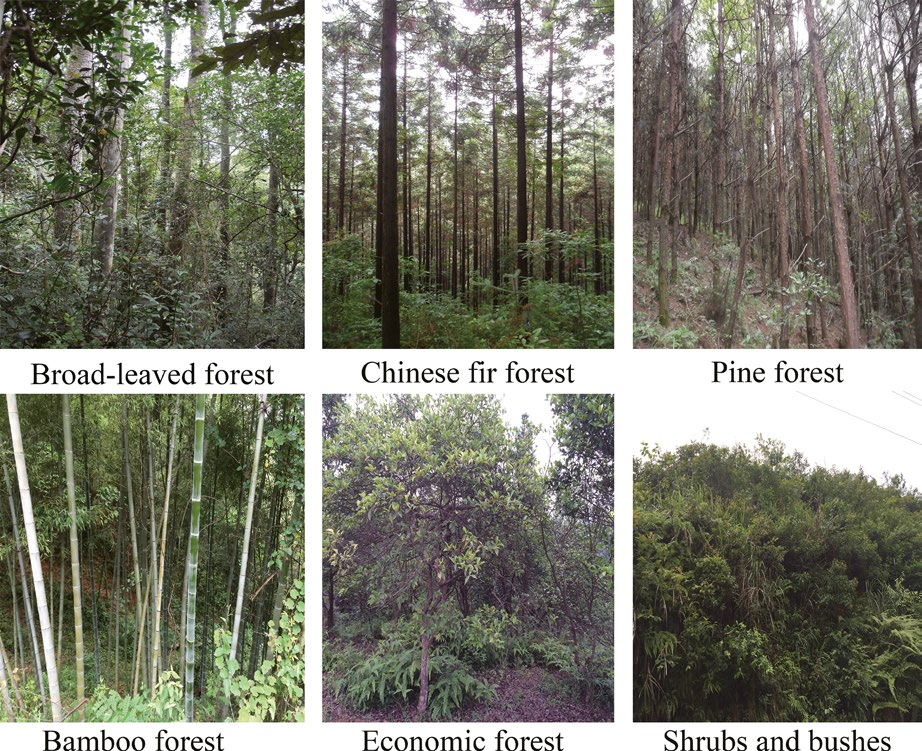
Field pictures of typical forests in the Gan River Basin

For carbon storage in forests in the GRB, the InVEST model aggregates the amount of carbon stored in four carbon “pools,” including aboveground biomass, belowground biomass, soil, and dead organic matter, based on the forest distribution map and carbon density data from the 6th forest resource inventory (2000–2005) (Wang & Wei, 2007). The forest distribution map in the GRB was determined by visual interpretation of the TM images in 2010 (http://eds.ceode.ac.cn). Forest resources in the GRB were divided into six types based on different dominant tree species including the Pine forest, the Chinese fir forest, the broad‐leaved forest, the bamboo forest, the economic forest, and the shrubs and bushes (Figure 3), and the accuracy of the remote sensing interpretation was tested using 2671 data plots from the 7th forest resource inventory (2005–2010). The economic forest in this study refers to artificial forests, including the *Camellia Oleifera* forest, the *castanea mollissima* forest and the sumac forest, used for producing nontimber forest products.

For the annual water yield in the forest, the InVEST model uses the Budyko curve and annual average precipitation to determine the annual water yield in each pixel. The average annual reference precipitation data and average annual reference evapotranspiration data are based on interpolation of meteorological data from the study area between 2000 and 2010 (http://cdc.nmic.cn/home.do). The depth to the root‐restricting layer data and the plant available water fraction data are from the Data Center for Resources and Environmental Sciences, Chinese Academy of Sciences (RESDC) (http://www.resdc.cn). The plant evapotranspiration coefficients for each tree species are from the FAO (http://www.fao.org/docrep/X0490E/x0490e0b.htm).

For the annual soil retention in the forest, the InVEST model computes the amount of eroded sediment using the revised universal soil loss equation (RUSLE) at an annual time scale and the amount of sediment eroded in the catchment and retained by vegetation and topographic features. In this process, the soil erodibility index was calculated according to the Williams equation (Williams & Arnold, 1997) based on soil texture data from the RESDC, and the rainfall erosivity index was calculated according to the Wischmeier method (Wischmeier & Smith, 1965) based on meteorological data from the China Meteorological Data Sharing Service System (http://cdc.nmic.cn/home.do). DEM data were obtained from the Geospatial Data Cloud (http://www.gscloud.cn/).

For the wood volume in the GRB, the CASA model and ArcGIS software were used. First, we computed the vegetation net primary productivity (NPP) using the CASA model based on NDVI data from Vision on technology (http://free.vgt.vito.be) and monthly precipitation, temperature, and solar radiation data from the China meteorological data sharing service system. Then, we evaluated the wood volumes of different forests based on the transformation equations presented in related studies conducted by Fang and Zeng (Fang, Liu, & Xu, 1996; Zeng, 2012).

Furthermore, we used the volume data in 2010 investigated by the 7th forest resource inventory (790 plots), the runoff depth data in 2010 measured by the hydrologic station based on river level (46 stations), and the sediment yield data in 2010 measured by the hydrologic station of the China's Ministry of Water Resources (10 stations) to test the evaluation accuracies of the four ecosystem services based on the Pearson double‐tail testing method through SPSS19.0 software.

### 2.3 Overall benefits and trade‐offs of ecosystem services

A simple statistical approach based on Bradford and D'Amato (2012) was used to quantify the overall benefits and trade‐offs among the four forest ecosystem services. This approach extends the meaning of trade‐offs from negatively correlated relationships (i.e., traditional sense) to the inclusion of uneven rates of same‐direction changes between ecosystem services (Lu et al., 2014). It is also a simple, effective way to routinely calculate the benefits and trade‐offs between any two or more ecosystem services, no matter how they are correlated (Lu et al., 2014).

Wood production is an important component of economic development in the GRB, and plantations, mainly Chinese fir plantations and Masson pine plantations, are the major timber suppliers. Therefore, based on the difference of ecosystem functions and wood productions between six forest types, the weight coefficients of different ecosystem services in different forest types are established (Table 1). Generally, provisioning services are products that people directly obtain from ecosystems such as food, water, timber, and regulating services are that could affect climate, floods, disease, and water quality (MA, 2005). Thus, the four forest ecosystem services in the GRB were divided into two groups. The first group was the provisioning services, including the wood volume and water yield, and another group was the regulating services, including the carbon storage and soil retention. Compared to the regulating services, provisioning service of planted forests was paid more attention in the GRB, and therefore volume service weight of the Pine forest and the Chinese fir forest which was mainly artificial forests was bigger than that of natural forests including the broad‐leaved forest, the bamboo forest and the shrubs and bushes. Considering that there are very few logging activities in the economic forest, equal weight was given to the provisioning service and regulating services. The benefit associated with a single forest ecosystem service in each pixel is calculated by multiplying the relative standardization value by the weight index based on the forest type. The individual ecosystem service benefit in each pixel ranges from zero to its weight index and can be conceptualized as an indicator of the service contribution in the cell.

**Table 1:** Ecosystem service weight coefficients of different forest types

Forest	Wood volume	Carbon storage	Water yield	Soil retention
Pine forest	0.4	0.2	0.2	0.2
Chinese fir forest	0.4	0.2	0.2	0.2
Broad‐leaved forest	0.25	0.25	0.25	0.25
Bamboo forest	0.25	0.25	0.25	0.25
Shrubs and bushes	0.25	0.25	0.25	0.25
Economic forest	0.25	0.25	0.25	0.25

The overall benefit associated with the four forest ecosystem services in each pixel can be estimated by summing the individual benefits, and the trade‐offs among the four ecosystem services in each pixel is a measure of benefit variation. One simple approach for quantifying the magnitude of the trade‐off between more than two ecosystem services is to calculate the root mean square error (RMSE) of the individual benefits (Bradford & D'Amato, 2012). Additionally, to compare different pixels, we calculated the coefficient of variation between the four benefits in each pixel instead of the RMSE.

### 2.4 Correlation and cluster analyzes

Physical geography and administrative levers are two important dimensions and operational spaces for forest management decision making (Bai et al., 2013; Turner et al., 2014). In the GRB, the important physical geography units include the subwatershed and patch geography. The former indicates the terrain and available water conditions, and the latter encompasses the basic components of the landscape pattern. State‐owned forest land accounts for 88.57% of the area in the GRB, and the main forest administrative units are state forest farms in each county. Service correlations (Pearson's *r*) between the four forest ecosystem services at pixel size (30 m × 30 m), patches, subwatersheds, and counties in the GRB were calculated through SPSS19.0 software to determine the most appropriate forest management units and reveal the interactions between the ecosystem services (Bai, Zhuang, Ouyang, Zheng, & Jiang, 2011; Chan et al., 2006; Egoh et al., 2011). Then, we classified some forest management zones based on hierarchical cluster analysis method through SPSS19.0 software with taking the mean values of the overall benefits, trade‐off covariance and the four ecosystem services in the most appropriate spatial units as input variables (Bai et al., 2011). This classification will help forest farm administrators scientifically establish management objectives in different counties.

## 3 RESULTS

### 3.1 Distribution of services

The forest area precision of the remote sensing interpretation was 88.35%, and that the simulated values of the carbon storage service were consistent with previous research results (Li, Shao, & Liu, 2012; Wei, Wang, & Guo, 2008), the simulated and observed values of the water yield service and wood volume service were significantly correlated (*R* ^2^ = 0.791, *p *=* *.01 and *R* ^2^ = 0.617, *p *=* *.01), and that of the soil retention service was less significantly correlated (*R* ^2^ = 0.479, *p *=* *.01).

Area of the Pine forest was the largest in the GRB and mainly distributed in the eastern regions with low altitude (Figure 1). Area of the Chinese fir forest was also larger than other forests, and mainly distributed in high altitude regions around the mountains in the GRB (Figure 1). Area of the other forest types in the GRB was small and showed spatially discrete distribution (Figure 1). Due to the extensive distribution areas, significant contributions of the four forest ecosystem services in the basin were found in the Pine forest and the Chinese fir forest. Other forests displayed contributions smaller than 1/10 of these contributions, except the Broad‐leaved forest, which is also important for four ecosystem services, especially the carbon storage service (Table 2). Total amount of carbon storage in the Broad‐leaved forest was similar to that in the Pine forest, although its distribution area is only approximately 1/3 that of the Pine forest. Thus, replacing the Broad‐leaved forest in the basin will greatly damage the carbon storage service and further influence regional climate stability because carbon storage helps mitigate the greenhouse effect.

**Table 2:** Total percentage (%) and production capacities of each ecosystem service in different forests

Forest	Area (%)	Carbon storage (%)	Carbon density (t/ha)	Wood volume (%)	Wood volume (m^3^/ha)	Water yield (%)	Water yield depth (mm)	Soil retention (%)	Soil retention (t/ha)	Soil erosion modulus (t/ha)
Pine forest	40.6	34.12	113.68	36.91	38.22	44.24	1475.52	40.76	457.36	21.85
Broad‐leaved forest	13.95	32.34	171.1388	15.04	67.59	10.76	1052.01	14.11	461.21	7.12
Chinese fir forest	32	17.65	136.71	39.67	51.32	32.81	1391.13	32.28	459.29	29.73
Bamboo forest	6.08	7.69	171.21	6.36	53.64	6.33	1417.01	6.19	463.97	22.17
Bushes and shrubs	0.95	7.3	127.51	0.29	19.6	1.04	1478.38	1.05	509.38	32.98
Economic forest	6.42	0.9	157.48	1.73	19.11	4.82	1039.62	5.6	407.09	49.29

**Figure 4:**
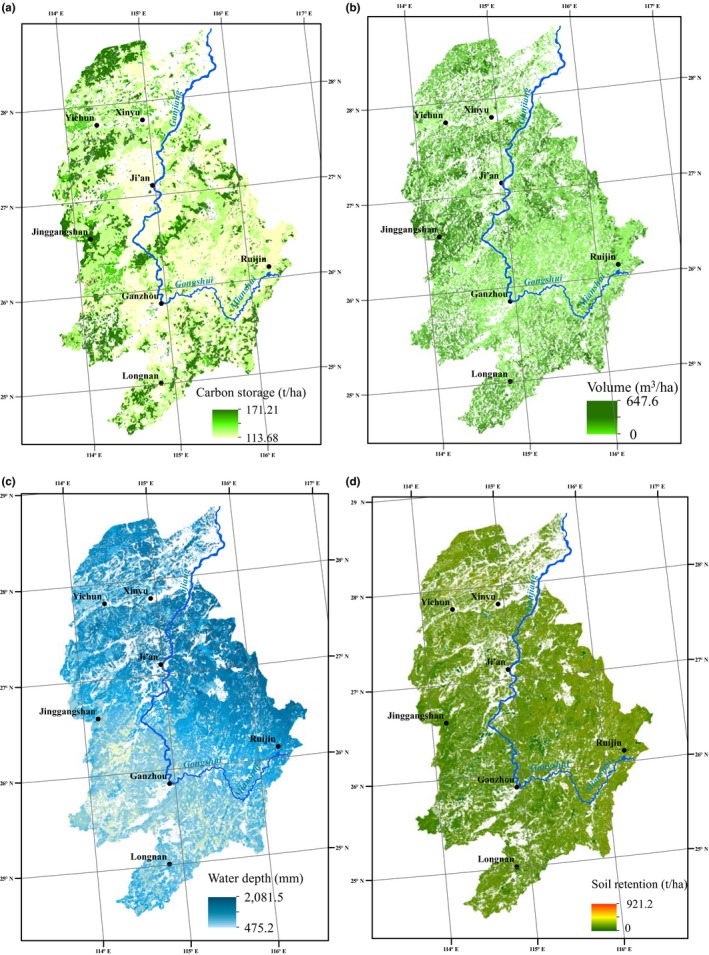
Spatial distributions of the four ecosystem services (a, carbon storage; b, wood volume; c, water yield; d, soil retention)

The spatial distributions of the four forest ecosystem services were also distinctly different (Figure 4). Due to high elevations and low human disturbance levels, broad‐leaved forests distributed in the northern regions of Yichun city, in the western portion of Ganzhou city, and around Jinggangshan city and Longnan city provided important carbon storage and wood volume services. Benefiting from the lower elevation and humid climate, forests in the north and the southeast regions of the basin were provided important water yield services, while forests that provided larger soil retention services were spatially scattered. The spatial heterogeneities between the four forest ecosystem services were different from the forest spatial distribution, suggesting that forest type variations in one region may not help obtain the expected response associated with the balance between providing ecosystem services and regulating services. Thus, it is better to integrate the important environmental factors into the management decision‐making process and utilize zone management based on the spatial heterogeneities of the ecosystem services.

### 3.2 Overall benefits and RMSE values of services

**Figure 5:**
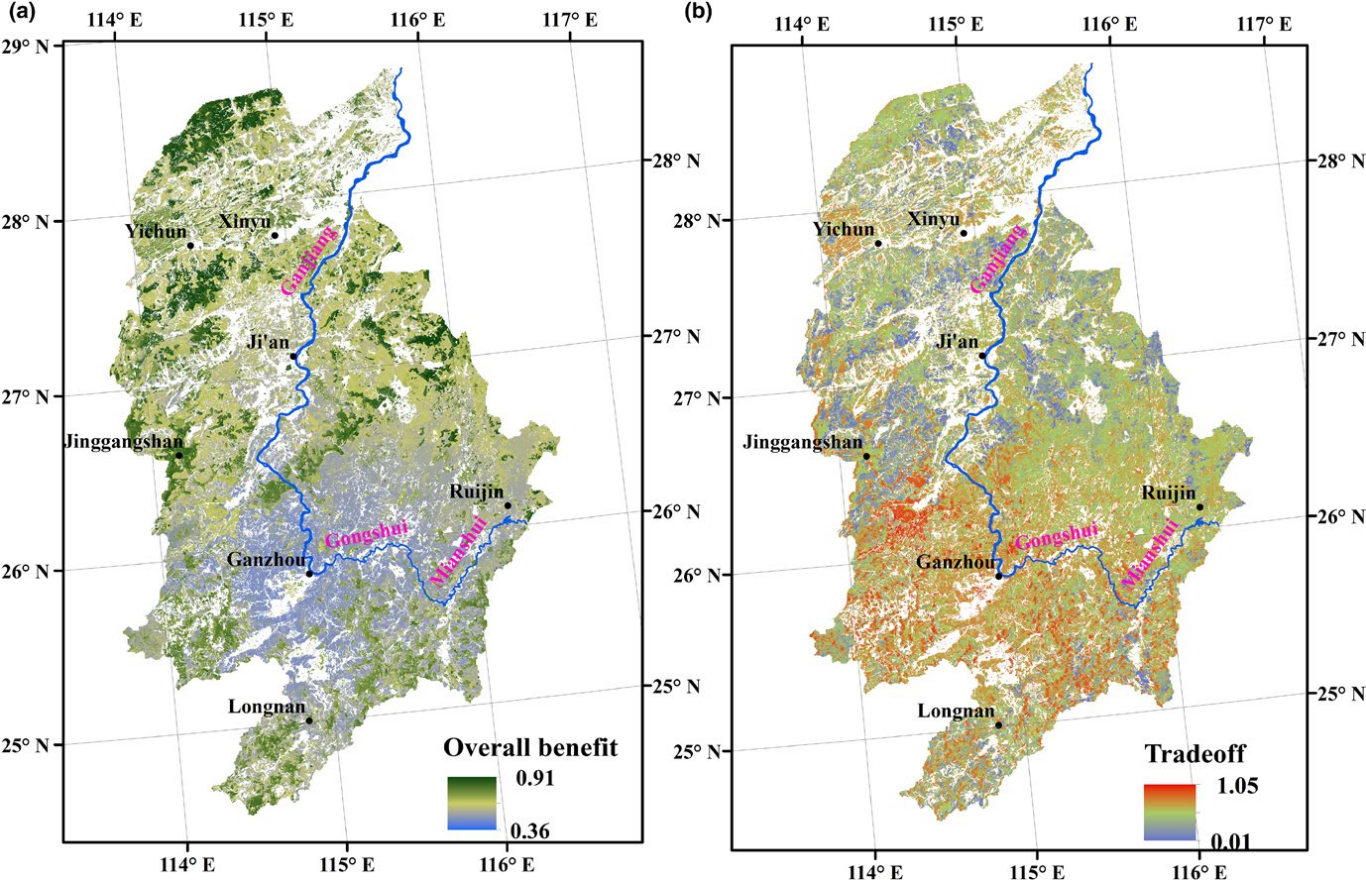
Spatial patterns of the overall benefits (a) and the root mean square error values (b) of forest ecosystem services in the Gan River Basin

The overall benefit of the forest ecosystem services was unequally distributed in space (Figure 5a). Forests scattered in mountainous areas around the basin displayed the highest benefit values. These forests were dominated by the broad‐leaved forest and the bamboo forest. Planted forests in downstream regions (as well as in the northern regions of the basin) showed higher overall benefits than those in upstream regions (as well as in the southern regions of the basin), and forests that showed the lowest overall benefits were dominated by pine forests.

In terms of the spatial patterns of service benefits based on RMSE values (Figure 5b), forests dominated by Chinese fir forests or pine forests in the middle reaches of the basin exhibited the lowest RMSE values, suggesting that there is little benefit difference between the four individual services. Forests dominated by broad‐leaved forests or economic forests in the southwestern portion of the upstream regions exhibited the largest RMSE values. Because of the poor environmental conditions, these forests exhibited lower water yields and wood volumes than did forests in other regions. The spatial patterns of service benefits based on RMSE values were not completely consistent with the pattern of the overall forest benefits (Figure 5a). Forests in mountainous areas around the GRB displayed the largest overall benefits; however, they did not exhibit the lowest RMSE values due to possessing a lower water yield than the water yield in planted forests. Forests dominated by economic forests and Chinese fir forests in the upstream regions displayed both the lowest overall benefits and RMSE values, indicating that benefit trade‐offs in these forests are substantial and should be given more attention.

Based on the statistical characteristics of the overall benefits in different forest types, the mean value of the overall benefit of the bamboo forest was the largest, and that of the broad‐leaved forest was the second largest (Table 3). The mean value of the overall benefit of the bushes and shrubs was similar to that of the economic forest and is at an intermediate level. Compared to natural forests, the mean value of the overall benefit of the Pine forest and the Chinese fir forest was at the lowest level and displayed a wide gap. Note that the range of the overall benefit of the Pine forest and the Chinese fir forest was wider than that of natural forests. Additionally, the maximum value was similar to that of natural forests, and the minimum value was much smaller, indicating that ecosystem service benefits displayed significant spatial heterogeneity in different plantation patches, with ample room for improvement.

**Table 3:** Statistical characteristics of the overall benefits (abbreviated as B) and RMSE values (abbreviated as R) of ecosystem services in different forests in the GRB. B‐Min, B‐Max, and B‐Std.: minimum, maximum, and standard deviation of the overall benefits. R‐Min, R‐Max, and R‐Std.: minimum, maximum, and standard deviation of the RMSE values

Forest	Benefit	B‐Min	B‐Max	B‐Std.	RMSE	R‐Min	R‐Max	R‐Std.
Bamboo forest	0.68	0.55	0.91	0.03	0.57	0.13	0.69	0.05
Broad‐leaved forest	0.64	0.56	0.88	0.03	0.62	0.19	0.76	0.07
Bushes and shrubs	0.61	0.46	0.79	0.02	0.59	0.14	0.65	0.04
Economic forest	0.60	0.38	0.77	0.04	0.65	0.21	1.05	0.06
Chinese fir forest	0.51	0.37	0.83	0.03	0.50	0.02	0.67	0.09
Pine forest	0.49	0.38	0.87	0.04	0.54	0.01	0.67	0.10

Furthermore, in terms of the statistical characteristics of the RMSE values in different forests, the economic forest and the broad‐leaved forest exhibited the highest RMSE mean values (Table 3). The RMSE mean value of the bamboo forest was similar to that of the bushes and shrubs and was at a moderate level. Compared to other forests, the RMSE mean value of the pine forest and the Chinese fir forest was the smallest, indicating that benefit differences associated with individual ecosystem services between different forest patches were small in these two forest types. The range of RMSE values in the economic forest was the widest and exhibited the maximum and minimum values. Thus, there was significant benefit difference associated with individual ecosystem services between different economic forest patches in different regions. The maximum RMSE values of the pine forest and the Chinese fir forest were similar to that of natural forests and the minimum RMSE values were smaller, indicating that differences in individual service benefits between planted forest patches were larger than those of natural forests.

**Figure 6:**
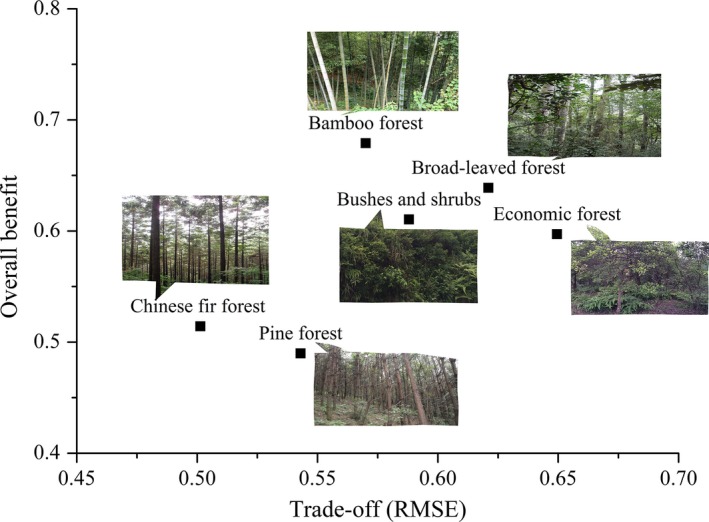
The overall benefit and trade‐off (RMSE) values in different forests

For effective forest management in GRB, forests with high overall benefits and stable or balanced relationships between provisioning and regulating ecosystem services should be built and increased. Based on Figure 6, the bamboo forest was the most suitable forest type to promote and increase in regions that are committed to developing forest by‐products. The broad‐leaved forest can also be increased due to high overall ecosystem service benefit, although inevitable trade‐off situations between wood production and water yield will occur. Although the pine forest and the Chinese fir forest were the main timber providers, their disadvantages were obvious because they provided very low overall benefits compared to other forests and even bushes and shrubs. Therefore, measures such as increasing middle‐aged plantations or extending the rotation interval will be attractive and necessary to improve plantation overall benefits based on their large planting areas.

### 3.3 Service trade‐offs and synergies

Relationships between the four forest ecosystem services were different at different spatial scales (Table 4). Two significant relationships were found at the pixel size. One was a synergy between the carbon storage service and the wood volume service. The other was a trade‐off between the carbon storage service and the water yield service. Other trade‐offs or synergies between ecosystem services were not significant. Relationships between forest ecosystem services became more significant at the patch level than that at the pixel size (Table 4). There were significant synergies between the carbon storage service and wood volume service, soil retention service and water yield service, as well as wood volume service and water yield service. Additionally, there was a significant trade‐off between the carbon storage service and water yield service. At the subwatershed scale, the correlation coefficients between four forest ecosystem services further increased compared to that at the patch lever (Table 4). Synergies between the carbon storage service and wood volume service, as well as soil retention service and water yield service, were more significant. Synergies between the wood volume service and water yield service became less significant. The trade‐off between the carbon storage service and water yield service became less significant and statistically insignificant. Furthermore, a new synergy between the carbon storage service and soil retention service was observed; however, it was not significant.

**Table 4:** Correlation coefficients between the four ecosystem services at different spatial scales

Spatial scale	Service	Carbon storage	Soil retention	Wood volume	Water yield
Pixel	Carbon storage	1			
	Soil retention	−0.00283	1		
	Wood volume	0.12638^a^	0.00083	1	
	Water yield	−0.43971^a^	0.06342	0.03755	1
Landscape	Carbon storage	1			
	Soil retention	−0.026	1		
	Wood volume	0.256^a^	0.061	1	
	Water yield	−0.142^a^	0.265^a^	0.133^a^	1
Subwatershed	Carbon storage	1			
	Soil retention	0.026	1		
	Wood volume	0.523^a^	0.1	1	
	Water yield	−0.066	0.717^a^	0.229^b^	1
County	Carbon storage	1			
	Soil retention	−0.151	1		
	Wood volume	0.401^a^	0.192	1	
	Water yield	−0.179	0.579^a^	−0.127	1

a Significantly correlated at the 0.01 level (bilateral).

b Significantly correlated at the 0.05 level (bilateral).

At the administrative county scale, relationships between the four forest ecosystem services changed slightly compared to that at the subwatershed scale (Table 4). There was still a significant synergy between the carbon storage service and wood volume service, as well as the soil retention service and water yield service; however, their correlation coefficients decreased slightly. Synergy between the wood volume service and the water yield service became less significant and statistically insignificant. The other relationships between ecosystem services became more significant, but they remained statistically insignificant.

### 3.4 Forest management based on service benefits

**Figure 7:**
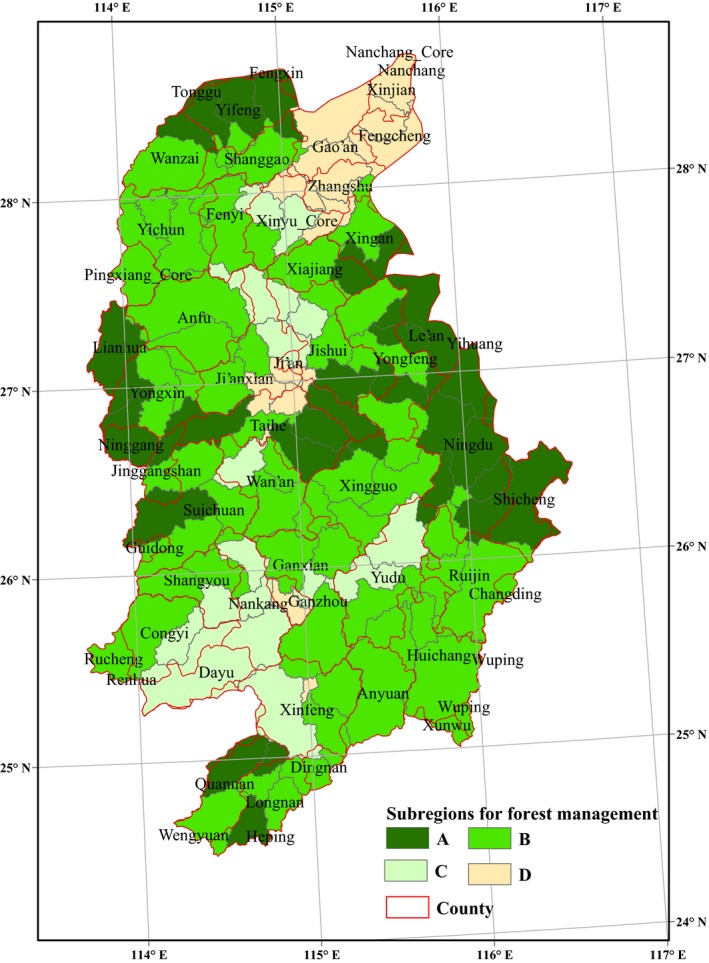
Subregions for forest management based on ecosystem service benefits. Forests in subregion A exhibited the largest overall benefits, forests in subregion B exhibited high overall benefits, forests in subregion C displayed moderate overall benefits, and forests in subregion D exhibited low overall benefits

The spatial patterns of individual ecosystem service benefits displayed heterogeneity because of forest type and environmental condition differences. To ensure that forest management decisions achieve both large overall benefits and balance individual ecosystem service benefits, it is better to combine the most significant trade‐offs or synergies between ecosystem services into forest management strategy and promote subregion or classified management based on the above spatial characteristics of the ecosystem services. A cluster analysis of the overall benefit of ecosystem services and RMSE values in 101 subwatersheds indicated that they could be divided into four subregions (Figure 7). Subregion A mainly comprises mountainous regions distributed along the eastern, western, and northern edges of the GRB, encompassing 24.53% of the basin area. Forests in subregion A exhibited the largest overall benefits and were composed of broad‐leaved forest, bamboo forest and small amounts of Chinese fir forest. The administrative counties in this subregion include Yi Feng, Lian Hua, Ning Gang, and so on. Subregion B occupies approximately half of the regions in the basin and is largely distributed from north to south across the interior of the GRB. Forests in subregion B exhibited high overall benefits and were dominated by pine forest and Chinese fir forest. The administrative counties include Yi Chun, Ping Xiang, Rui Jin, Xing Guo, and so on. Subregion C is located in the southwest and north central regions of the GRB, encompassing 13.97% of the basin area. Forests in subregion C displayed moderate overall benefits and were dominated by pine forest and economic forest. The administrative counties in subregion C include Nan Kang, Xin Yu, Yu Du, Ji'an, and so on. Subregion D is very small and distributed in the central and northeast regions of the GRB, including the counties of Gao'an, Zhang Shu, Xin Jian, Feng Cheng, and so on. Forests in subregion D are mainly dominated by pine forest and economic forest, with low coverage rates and spatial dispersion. Thus, they exhibited low overall benefits.

## 4 DISCUSSION

### 4.1 Service benefits in different forests

Spatial patterns of the four forest ecosystem services in the GRB showed heterogeneity because of production capacity differences associated with the individual ecosystem services from place to place. The carbon storage service and wood volume service in mountainous areas were higher than those in other regions (Figure 4) because forests in these regions were farther from human disturbances and characterized as late‐successional or high‐density canopies. The water yield services in plains distributed in the northeast region were high due to the flat terrain and humid climate. This area is also characterized by considerable human activities, and forests were dominated by forest plantations. Spatial patterns of the soil retention service were more evenly distributed, except for a few regions dominated by the economic forest and the bushes or shrubs. Thus, factors influencing forest ecosystem services are very complicated, and the relative importance of different factors is different for different services, creating a very difficult forest management situation. In addition, different economic benefits between providing services and regulating services also complicate forest management decision making (Costanza, 2008; Fisher, Turner, & Morling, 2009; de Groot, Alkemade, Braat, Hein, & Willemen, 2010).

Planted forests in the GRB were identified to be the largest providers of the four forest ecosystem services due to their wide distribution (Table 2). Although planted forests have large economic benefits due to timber products, compared to natural forests, the economic benefit of timber output from planted forests is due to a large total amount rather than a quality advantage and to the carbon storage service and the soil retention service (Table 2). Thus, except for the water yield service, the production capacities of the other ecosystem services and the overall benefits of plantations were lower than those of natural forests. Additionally, the production capacities of individual ecosystem services in planted forests varied from place to place, especially in the economic forest, which displayed the poorest soil retention service. Thus, many planted forest patches may have soil and water loss problems. Note that small parts of planted forests in the northern central regions of the basin exhibited high overall benefits associated with ecosystem services, approaching that of natural forests. Therefore, planted forests with similar environmental conditions can improve their overall ecosystem service benefits by learning from the successful management approaches of the above‐mentioned plantations.

### 4.2 Service trade‐offs and synergies with forest management

Different ecosystem services are related to different ecological processes at different scales. Thus, ecosystem service interactions will vary and trade‐offs or synergies between ecosystem services will change due to different spatial scales (Burkhard, Kroll, Nedkov, & Müller, 2012; Fisher & Turner, 2008). By comparing the correlation coefficients between the four forest ecosystem services in the GRB in the context of natural and administrative regionalization, we identified some significant relationships between local ecosystem services at the subwatershed scale. Significant synergy was found between the carbon storage service and the wood volume service, as well as between the water yield service, the wood volume service and the soil retention service. Additionally, important trade‐offs between the carbon storage service and the water yield service were observed. Thus, forest management measures that improve plantation structure and increase timber production will also improve regulating service production and the overall benefits of ecosystem services based on these relationships.

The 101 subwatersheds in the GRB were divided into four subregions. This division will be helpful in analyzing relationships between ecosystem services and human activities (Martin‐Lopez et al., 2012; Prato, 2012; Rodriguez et al., 2006). Subregion A, which is located in the mountainous regions along the edges of the north central part of the basin, showed high ecosystem service benefits as the result of late‐successional forests, good climate conditions, and ample precipitation compared with arid environments. Thus forest management measures in this region tend to have high‐priority for conservation. Subregions (subregion C and subregion D) distributed in the northeast and the southern portions of the basin displayed low forest ecosystem service benefits due to low forest coverage rates and high intensities of human activities, and so forest management measures tend to be used to improve the regulating services in order to enhance the urban landscape greening. Most forests (subregion B) dominated by planted forests exhibited similar benefits, although their site conditions are not consistent, suggesting that a majority of plantations have not made full use of advantageous environment conditions and that forest management is lacking based on regional characteristics. Forest management measures such as increasing the mid‐maturation plantations, artificial inducement, will be greatly preferred in this region.

The trade‐offs and synergies between ecosystems services at different spatial scales suggest that forest management should pay attention to the holistic theory involving ecosystem services and balanced relationships between providing services and regulating services based on local environmental problems to improve the overall benefits rather than the economic benefit of a single service. Higher soil erosion rates were identified in planted forests (the pine forest, the Chinese fir forest, and the economic forest) than in natural broad‐leaved forests (Table 2). Thus, based on scientific evidence, it is necessary to transform some low quality forest plantations into secondary natural forests to offset soil loss problems in the red soil hilly regions distributed in the southern portion of the basin. In addition, forest management subregions cross administrative boundaries, suggest that regional cooperation and negotiation will be essential and inevitable when implementing forest management strategies.

### 4.3 Limitations

Forest ecosystems provide goods and services closely related to the needs of social and industrial production. These goods and services are influenced by ecological processes at different spatial scales (Burkhard et al., 2012; Fisher & Turner, 2008). Spatially quantifying and mapping these forest ecosystem services will shed light on scale effects and interactions between ecosystem services, as well as build a scientific foundation for forest management decision making (Bennett & Balvanera, 2007; Pergams & Zaradic, 2008; Tallis, Kareiva, Marvier, & Chang, 2008; Van Wilgen & Richardson, 2014). While we scientifically analyzed and mapped the main forest ecosystem services in the GRB using the InVEST and CASA models, our study is nevertheless constrained by the limited availability of data.

One shortcoming was the ecosystem service evaluation accuracy. We used available hydrological station data, field‐sampled forest data, and relevant literature data (above‐mentioned) to test the accuracies of the four studied services. Because the carbon density data are not the latest, the total carbon storage in the GRB will be smaller than the actual value, and the spatial accuracy can be further improved if forest age composition and carbon density data are available. The assessments of water yield and wood volume were more accurate than that of carbon storage service based on the statistically significant correlations between the simulated and observed values. Evaluation of the soil retention service yielded a relatively low accuracy because that the sediment data from the China's Ministry of Water Resources was a mean value of a small watershed near a hydrological station, while the predicted value was a mean value of a pixel. If more detailed river map were offered, the accuracy of the soil retention service will be higher as that of the predicted value (56.95 t/ha) on the whole basin was similar to the observed value (59.8 t/ha) from of that. In addition, the InVEST model is widely controversial because of its low spatial accuracy and simple mechanisms (Bai et al., 2011; Sánchez–Canales et al., 2015). Its services evaluation approach and parameters need to be modified based on the environmental characteristics in specific regions (Chan et al., 2006; Onaindia et al., 2013; Turner et al., 2014).

The second shortcoming is related to the details of the analysis of overall ecosystem service benefits and interactions. When we computed the overall benefits of the four services in different forest types, weight values of each individual service had been allocated based on their linkage with human society and their management objectives. Because it is often ineffective to use service supplies to represent service economic outputs (Burkhard et al., 2012; Fisher & Turner, 2008), especially for regulating ecosystem services, weight values associated with the regulating services in our study, including water yield, carbon storage, and soil retention, may be too large, resulting in lower importance values in planted forests. Because forests are complex and dynamic systems, the key ecological process and by‐products have not been completely determined. Thus, calculating and allocating the overall benefits and weight values of many services will require more evidence and theory from environmental mechanism research rather than being based on economic indicators.

## 5 CONCLUSION

Our study indicates that natural forests and planted forests have significantly different ecosystem service productions and interactions. Taking the ecosystem service characteristics of different forest types into account can optimize forest management strategies and benefit spatial resource allocation. The primary lessons and recommendations arising from this study are as follows:

1. Benefiting from a wide distribution, planted forests in the GRB are the main ecosystem service producers, even though they exhibited low volume accumulation and soil retention per hectare, resulting in water loss, soil erosion, and pollution problems. Compared to planted forests, natural forests such as the broad‐leaved forest and the bamboo forest made larger contributions per hectare and were more important for regulating services, including carbon storage and soil retention services. Planted forests were more important for the water yield service.
2. Natural forests in mountainous areas around the GRB displayed the largest overall benefits and middle‐level benefit trade‐offs due to lower human disturbances and late‐successional forests. Planted forests in the middle portions of the upstream regions exhibited the lowest overall benefits and weak trade‐offs because of severe water and soil loss problems and poor forest quality. Based on the regulating service advantages of natural forest types, increasing the broad‐leaved forest area or recovering native vegetation in upstream regions in the GRB will improve environmental protection and local economic development.
3. Statistically significant interactions between ecosystem services were identified at different spatial scales, and relationships between regional regulating ecosystem services such as wood volume, water yield, and soil retention were more significant in landscape units and subwatersheds than that in administrative units or at a particular cell size. Therefore, forest management should account for the spatial heterogeneities of different ecological processes and their linkages with different services during decision making. Forest or natural resource management practices that ignore key regional environmental processes will lead to failure associated with the project targets.
4. An effective forest management scheme based on the most significant interactions between provisioning services and regulating services in the GRB was designed. Natural forest resources in mountainous areas in the GRB should be protected and restrictedly used to maintain regional biodiversity and water conservation. Planted forests in the northeast part of the GRB are the main providers of wood production. Thus, management strategies should pay more attention to structure transformation and wood volume improvement, as well as avoiding and diminishing soil erosion. Forests in the upstream regions exhibited poor environmental conditions and very low productivity. Therefore, management strategies should consider converting the plantations to native vegetation and promoting ecological restoration projects.

## CONFLICT OF INTEREST

None declared.
